# Predictive power of combined inflammatory markers and magnetic resonance imaging features for glioma grading using machine learning: a retrospective study

**DOI:** 10.1186/s12880-025-01946-0

**Published:** 2025-10-21

**Authors:** Yaohua Liu, Yuting Wang, Junle Zhu, Shuang Qin, Yi Hong, Saifei Sun, Qin Zhang, Qi Lv

**Affiliations:** 1 Department of Pediatrics, Tongji Hospital, Tongji University School of Medicine, Xincun Road No. 389, Shanghai, China; 2 Department of Medical Imaging, Tongji Hospital, Tongji University School of Medicine, Xincun Road No. 389, Shanghai, China; 3 Department of Neurosurgery, Tongji Hospital, Tongji University School of Medicine, Xincun Road No. 389, Shanghai, China; 4 Department of Clinical Research Center, Tongji Hospital, Tongji University School of Medicine, Xincun Road No. 389, Shanghai, China; 5 Department of Medical Imaging, Fudan University Affiliated Xuhui Hospital (Shanghai Xuhui Central Hospital), 366 Longchuan North Road, Xuhui District, Shanghai, 200031 China

**Keywords:** Inflammatory markers, Magnetic resonance imaging, Glioma grading, Machine learning

## Abstract

**Background:**

This study aimed to explore the potential of integrating magnetic resonance imaging (MRI) features with inflammatory markers to predict glioma grading using machine learning models.

**Methods:**

A total of 179 glioma patients were analyzed. The dataset was randomly split into a training set (75%) and an independent test set (25%) to test hyperparameter. To enhance reliability, five-fold cross-validation was also applied during the training phase. Key MRI features and inflammatory markers, including relative apparent diffusion coefficient (rADC), monocyte count, lymphocyte-to-CRP ratio (LCR) and hemoglobin-albumin-lymphocyte-platelet index (HALP), were extracted and used as inputs for multiple machine learning classifiers. Model performance was assessed using metrics such as area under the curve (AUC), accuracy, and F1 score.

**Results:**

The support vector machine model exhibited superior predictive performance, achieving an AUC of 0.92 and an F1 score of 0.91, effectively distinguishing between high-grade and low-grade gliomas.

**Conclusions:**

The combination of MRI features and inflammatory markers, analyzed through machine learning models like SVM, provides some clues for refining glioma prognosis and guiding personalized treatment strategies.

**Supplementary Information:**

The online version contains supplementary material available at 10.1186/s12880-025-01946-0.

## Background

Gliomas, the most common primary tumors of the central nervous system, present a significant clinical challenge due to their high recurrence rates and poor overall survival. This is further compounded by the limited availability and often suboptimal effectiveness of current treatment options [1]. Traditionally, glioma prognosis has relied on invasive procedures such as surgical biopsy and histopathological evaluation. However, the inherent heterogeneity of gliomas can hinder the accuracy of pathological assessments. Recent advances in medical imaging and tumor biology have facilitated the development of non-invasive methods for prognostic evaluation [2].

Magnetic resonance imaging (MRI) has become a cornerstone in the diagnosis of brain tumors, offering critical insights into tumor morphology and, to some extent, metabolic activity. Among the key MRI-derived metrics, the contrast enhancement ratio (ER) and the apparent diffusion coefficient (ADC) from diffusion-weighted imaging (DWI) are particularly significant. ER reflects tumor vascularity and aggressiveness, whereas ADC indicates tumor cellularity and malignancy potential. The relative ADC (rADC), defined as the ratio of the lesion-focused ADC to that of the surrounding tissue, serves as a normalized marker of water molecule diffusion, reducing the influence of inter-individual variability. Both ER and rADC are integral to glioma grading, prognosis, and treatment monitoring, offering valuable insights into tumor biology and behavior [3, 4].

Moreover, emerging evidence indicates that hematological markers of systemic inflammation can serve as reliable predictors in the preoperative assessment of cancer. Elevated markers such as the neutrophil-to-lymphocyte ratio (NLR) have been associated with poor outcomes in patients with neurogliomas [5, 6]. The rationale for combining systemic inflammatory markers with radiologic features in glioma stems from the understanding that gliomagenesis and progression are influenced by both local tumor characteristics and the systemic inflammatory response [7]. Prior studies have demonstrated that the interplay between tumor-induced inflammation and angiogenesis, as reflected by MRI parameters like ER, can synergistically predict glioma grade and prognosis [8]. For instance, a study showed that combining NLR with MRI-derived texture features improved the accuracy of glioma grading compared to using either modality alone [9]. These findings suggest that integrating inflammatory markers with imaging data can provide a more comprehensive assessment of the tumor microenvironment and its impact on disease progression.

In this context, a feasibility study has highlighted the potential of using advanced imaging techniques and machine learning algorithms to enhance the prognostic evaluation of glioblastoma, demonstrating that such approaches can significantly improve predictive accuracy and clinical decision-making [10]. However, limited research has investigated the combined predictive value of inflammatory and nutritional markers in the pathological grading of gliomas, particularly in the context of advanced machine learning techniques. Furthermore, the feasibility of using such a combined approach to predict outcomes in specific glioma subtypes, such as glioblastoma, warrants further investigation. A recent study explored the use of radiomic features derived from MRI in conjunction with clinical data to predict survival in glioblastoma patients, demonstrating the potential of integrating multi-modal data for improved prognostication [11]. This highlights the need for further research to refine and validate these approaches in larger, more diverse patient cohorts.

The selection of non-invasive biomarkers is critical for accurately predicting the pathological grade of gliomas. Machine learning models are particularly well-suited for this task, as they can effectively analyze complex, multidimensional datasets and reveal subtle relationships among imaging parameters, inflammatory markers, and tumor prognosis. Therefore, this study aims to use artificial intelligence technology to seek its sensitive indicators and evaluate predictive ability in glioma grading by comprehensive analysis of clinical data, imaging characteristics and inflammatory markers.

## Methods

### Study participants

This study was a retrospective single-center study with continuous enrollment of glioma patients treated in the neurosurgery department of Shanghai Tongji hospital between January 2022 and December 2024. All pathological and clinical data were collected and verified by experienced physicians. Glioma grading was conducted according to the 2016 World Health Organization (WHO) Classification of Tumors of the Central Nervous System. The study was approved by the hospital’s ethics review board, and written informed consent was obtained from all patients before participation.

### Inclusion criteria

The patient must be pathologically confirmed as having glioma (WHO grades I-IV); MRI imaging must be completed within three days prior to surgery with complete visual characteristics and data suitable for subsequent quantitative analysis; Comprehensive records of inflammatory markers and clinical features are required, with blood test results for inflammatory markers obtained within 48 hours before surgery; Complete follow-up data and survival outcome information must be provided.

### Exclusion criteria

Image quality does not meet the analysis requirements (such as strong motion artifacts, missing sequences, severe artifacts, etc.);

History of radiotherapy, chemotherapy or other interventions known to alter the tumor microenvironment;

Having any of the following conditions that may significantly affect levels of inflammatory markers and not excluded or stabilized prior to blood sample collection: a) Acute or persistent infections (e.g., active meningitis, cerebrospinal fluid infections, systemic bacterial/viral infections, etc., uncontrolled by non-therapeutic interventions during blood testing), b) Immune/inflammatory diseases (e.g., rheumatoid arthritis, systemic lupus erythematosus, inflammatory bowel disease, ankylosing spondylitis, etc.), c) Major trauma or surgical history within two weeks prior to blood sample collection, d) Recent use of systemic glucocorticoids, immunosuppressants, or long-term anti-inflammatory drugs (diagnosed within two weeks prior to blood testing), e) Pregnancy or delivery period, f) Coexisting malignancies or chronic liver/kidney dysfunction that may interfere with inflammatory markers (diagnosed within four weeks prior to blood testing), g) Other conditions that may significantly affect inflammatory marker levels or, as determined by investigators, potentially compromise study outcomes.

### Blood test

Peripheral blood samples were collected from all patients, and routine hematological analyses were performed. The measured or calculated parameters included white blood cell count, neutrophil count, lymphocyte count, platelet count, serum albumin concentration, and C-reactive protein (CRP) levels. Based on these values, several composite inflammatory and nutritional indices were derived, including NLR, derived NLR (dNLR), platelet-to-lymphocyte ratio (PLR), platelet-to-monocyte ratio (PMR), lymphocyte-to-monocyte ratio (LMR), lymphocyte-to-CRP ratio (LCR), neutrophil-to-CRP ratio (NCR), prognostic nutritional index (PNI), advanced lung cancer inflammation index (ALI), albumin-lymphocyte-platelet-CRP index (ALPC), systemic immune-inflammation index (SII), CRP-albumin-lymphocyte index (CAL), CRP/albumin ratio (CAR), and hemoglobin-albumin-lymphocyte-platelet index (HALP). The detailed formulas for calculating these indices are provided in the Appendix 1.

### MRI

MRI data were acquired using a 3.0T SIEMENS Verio superconducting magnetic resonance scanner. The imaging protocol included T2-weighted imaging (T2WI), unenhanced and contrast-enhanced T1-weighted imaging (T1WI), and DWI. The acquisition parameters were as follows: for T2WI, repetition time (TR)/echo time (TE) = 4210/96 ms; for T1WI, TR/TE = 1530/9 ms, with a pixel matrix of 256 × 256, field of view (FOV) of 23 × 23 cm^2^, slice thickness of 5 mm, and an intersection gap of 1 mm. For contrast-enhanced imaging, 3 mL/s of gadopentetate dimeglumine was administered intravenously, followed by contrast-enhanced T1-weighted image acquisition. DWI was performed with TR of 5400 ms, TE of 94 ms, and b-values of 0 and 1000 s/mm^2^. Axial, coronal, and sagittal planes were scanned for all sequences.

### Segmentation

This study primarily focuses on preoperative ET (Enhancing Tumor) as the main segmentation target. Edematous regions are identified using T2/FLAIR sequences as supplementary markers, with the entire tumor-edema boundary defined through ET delineation. The segmentation work in this study was performed using the hospital radiology PACS system. Two radiologists with experience in neurology radiology were compared after independent evaluation. If there was disagreement, the final consensus ROI was formed through consensus consultation. The segmentation tool utilized was the built-in ROI drawing/annotation function of the hospital PACS system, with ROI areas ranging from 15 to 50 mm^2^.

ER, rADC, and EI based on consensus ROI area were calculated by two physicians independently. The calculation results of the two radiologists were evaluated for intergroup consistency using the interclass correlation coefficient. When the ICC value is below 0.5, it indicates poor consistency strength in measurement results. An ICC value between 0.5 and 0.75 reflects moderate consistency strength. Values ranging from 0.75 to 0.9 demonstrate good consistency in measurement results. When the ICC value exceeds 0.9, it signifies excellent consistency strength.

### Feature selection and preprocessing

To minimize heterogeneity bias among patients, all data were standardized using the Min-Max normalization method. Feature selection was performed using the analysis of variance (ANOVA) F-test to identify features with significant heterogeneity. Additionally, the Pearson correlation coefficient was applied to evaluate the relevance between each feature and the target variable.

### Group classification

The selected features were used to predict glioma grades. Patients with grade 1 and 2 gliomas were categorized into the lower-grade group, whereas those with grade 3 and 4 gliomas were classified into the higher-grade group.

### Classifier building and assessment

Three classical machine learning classifiers—k-nearest neighbors (kNN), support vector machines with a radial basis function kernel (r-SVM), and random forest (RF)—were constructed and compared.

#### Hyperparameter tuning

The dataset was initially split into training and test cohorts at a ratio of 3:1. Using accuracy as the evaluation metric, we tested different hyperparameters: KNN model with nNeighbors = [1: 40], SVM model with C = i × 5/100 (i= [1: 40]), and RF model with nEstimators = [1: 100]. We selected hyperparameters that demonstrated stable accuracy fluctuations as the final criteria.

#### Model development

Following feature selection and hyperparameter tuning, the selected features were used as inputs to develop machine learning classifiers. Each model was evaluated using five-fold cross-validation to ensure robust performance. The average predictive performance across the validation sets was assessed using accuracy and the area under the receiver operating characteristic (ROC) curve (AUC). The confidence interval (CI) is calculated by Gaussian distribution algorithm. Model stability was further quantified using the F1-score. The calibration evaluation utilized Brier Score, log loss, expected calibration error (ECE), and reliability curves. Additional evaluation metrics included sensitivity, specificity, positive predictive value, and negative predictive value. ROC curves were generated based on the cross-validation results to visualize classification performance. To assess clinical relevance, overall survival (OS) and progression-free survival (PFS) were analyzed using the Kaplan–Meier method, with differences between groups evaluated via the log-rank test.

All statistical analyses and machine learning model development were performed using Python version 3.11. Survival analyses were conducted using R version 4.3.1.

## Results

### General information

A total of 179 patients with gliomas were included in the final analysis. Detailed demographic and clinical characteristics are summarized in Table 1. The mean age of the patients was 52.09 (range: 3–86) years. Based on the WHO classification, gliomas were distributed as follows: grade I (*n* = 10), grade II (*n* = 22), grade III (*n* = 35), and grade IV (*n* = 112). The ICC values were all greater than 0.9. The enhancement index (EI), rADC, and ER were selected from the calculations of one radiologist.

**Table 1:** Demographic and clinical characteristics of patients, along with ANOVA F-test results for feature selection

	Low-Grade Glioma	High-Grade Glioma	P value
Patients(n)	32	147	—
Age*	37.28(5–81)	55.31(3–86)	0.000
EI	2.75(1.15–6.6)	5.76(1.07–179.26)	0.283
rADC*	1.79(0.19–3.77)	1.43(0.7–2.87)	0.000
ER	1.48(0.87–3.14)	1.64(0.8–3.19)	0.118
WBC	6.46(4.03–10.7)	7.15(3.58–25.14)	0.207
N	3.93(1.99–8.71)	4.74(1.36–23.81)	0.139
L	1.95(1.1–3.55)	1.78(0.18–4.5)	0.216
Plt	229.59(142–357)	210.72(78–401)	0.118
M*	0.77(0.24–5.8)	0.47(0.1–1.11)	0.011
CRP	3.15(0.2–61.87)	5.72(0.2–218.02)	0.524
ALB	40.1(34.1–48.9)	39.04(26.4–50.4)	0.108
NLR	2.3(0.83–7.82)	4.08(0.52–132.28)	0.366
Dnlr	1.31(1.19–1.91)	1.42(1.09–7.39)	0.258
PLR	129.52(57.36–324.55)	141.9(34.36–1261.11)	0.548
PMR	558.34(24.48–900)	509.4(143.33–1883.33)	0.327
LMR	4.6(0.26–8.9)	4.17(0.16–14)	0.266
LCR*	5.47(0.02–17.75)	3.25(0–17.9)	0.008
NCR	8.55(0.14–18.2)	7.21(0.06–49.3)	0.436
PNI	49.85(39.6–62.35)	47.93(33.65–67.8)	0.063
ALI	559.54(98.1–1369.87)	465.29(6.63–2125.61)	0.136
ALPC	0.27(0–0.99)	0.24(0–1.41)	0.602
SII	565.47(170.97–2791.09)	881.67(75.69–30027.06)	0.480
CAL	0.07(0–1.65)	0.27(0–18.98)	0.536
CAR	0.09(0–1.81)	0.16(0–7.12)	0.543
HALP*	0.01(0–0.02)	0.01(0–0.09)	0.017

From age to HALP, the value stand for Mean(min-max)

p-values are reported for each feature based on the ANOVA F-test

^*^p value < 0.05 N: Neutrophil count L: Lymphocyte count M: monocyte count Every abbreviation has been annotationed in Supplementary Material 1.

### Feature selection

**Fig. 1:**
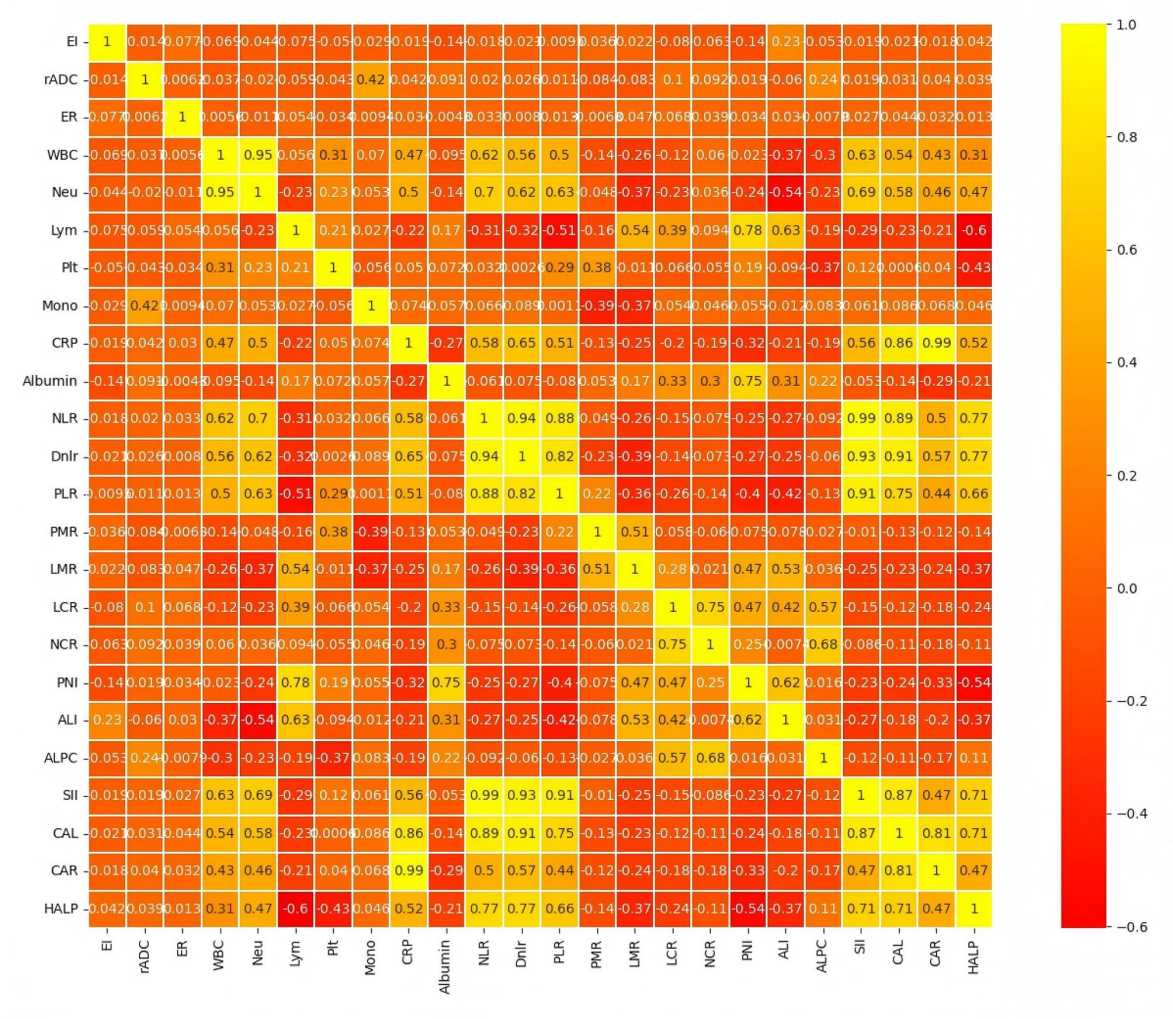
Pearson correlation coefficient analysis used for feature selection

Based on the ANOVA F-test results (Table 1), significant variability was observed in several variables, including age, rADC, monocyte count, LCR, and HALP. Additionally, Pearson correlation coefficient analysis (Fig. 1) revealed multicollinearity or weak correlation with the target variable for certain features, specifically WBC, dNLR, PLR, NLR, CAL, and CAR, which were subsequently excluded. Consequently, the following features were retained for model training: rADC, monocyte count, LCR, and HALP.

### Hyperparameter tuning

**Fig. 2:**
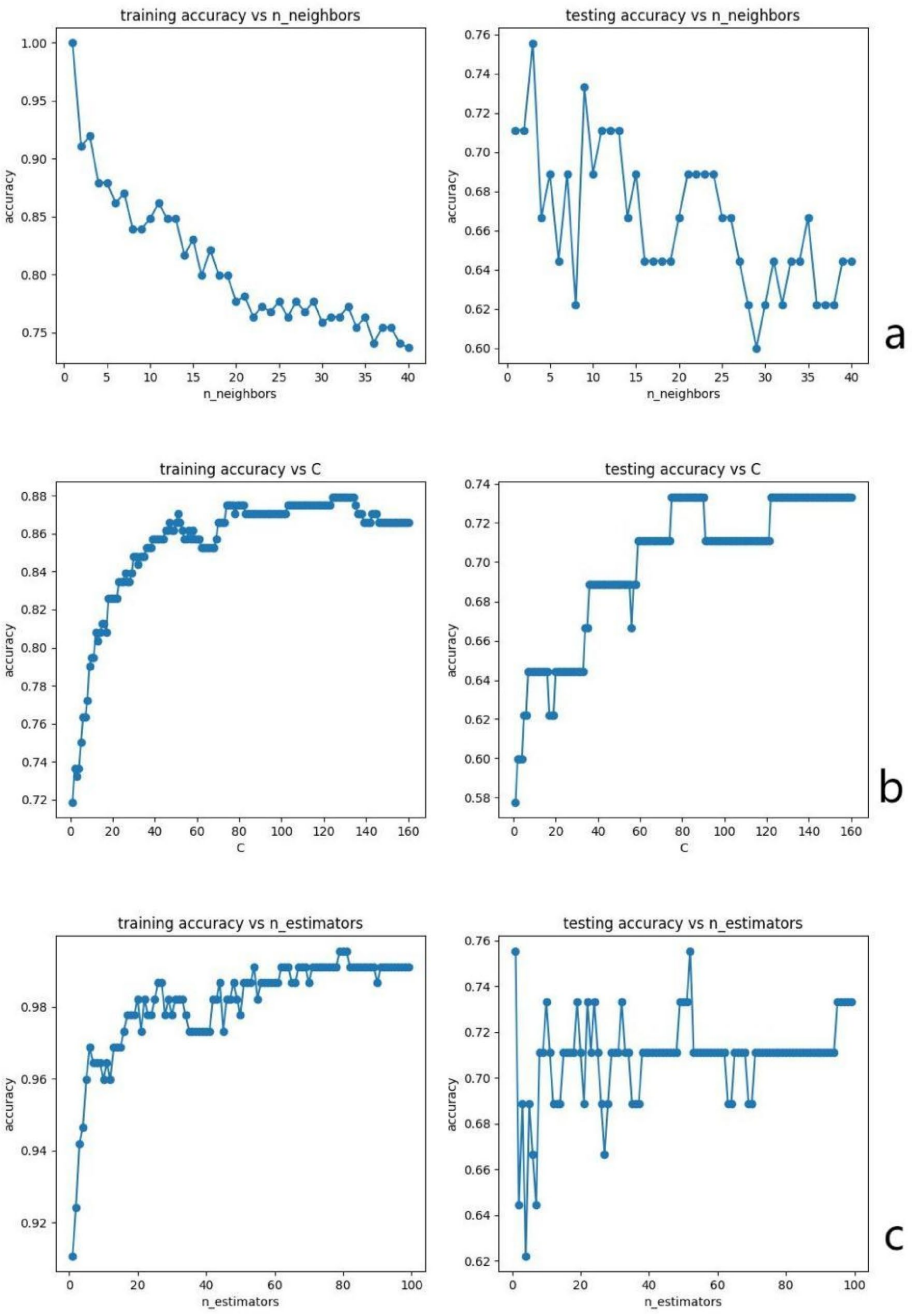
The relationship curve between model accuracy and variation of hyperparameters. (**a**) KNN(k-nearest neighbor classification); (**b**) r-SVM(support vector machine); (**c**) RF(random forest)

For the KNN model, the number of neighbors was set to *n_neighbors* = 4. In the RF model, the number of trees was set to *n_estimators* = 80, and the minimum number of samples required to split an internal node was set to *min_samples_split* = 5. For the SVM model, the regularization parameter was set to *C* = 4. All other hyperparameters were kept at their default values. Figure 2 shows the variation of accuracy with hyperparameters.

### Classification of low-grade (WHO I and II) and high-grade (WHO III and IV) gliomas

#### Evaluation of data feature differences between training and test cohorts

In the training and test cohorts used for 5-fold cross-validation, there were approximately 117 patients with high-grade glioma and 26 patients with low-grade glioma in the training cohorts, 30 patients with high-grade glioma and 6 patients with low-grade glioma in the test cohorts. The ANOVA F-test was conducted on the features between the training and test cohorts. Results are shown in the Table 2. Except for the fifth group HALP index *p* value was 0.04, the rest The *p* values were all greater than 0.05.

**Table 2:** Five-fold cross-validation cohorts: low-grade glioma vs. high-grade glioma

Turn	train_LGG	train_HGG	test_LGG	test_HGG	rADC_pVal	Mono_pVal	LCR_pVal	HALP_pVal
1	26	117	6	30	0.70	0.43	0.10	0.70
2	26	117	6	30	0.26	0.58	0.15	0.21
3	25	118	7	29	0.71	0.22	0.81	0.53
4	25	118	7	29	0.12	0.54	0.87	0.80
5	26	118	6	29	0.66	0.46	0.84	0.04

train: training cohort; test: test cohort; _LGG: LGG count; _HGG: HGG count; _pVal: p value of ANOVA F test between training and test cohorts

#### Evaluation of the predictive performance of models

**Fig. 3:**
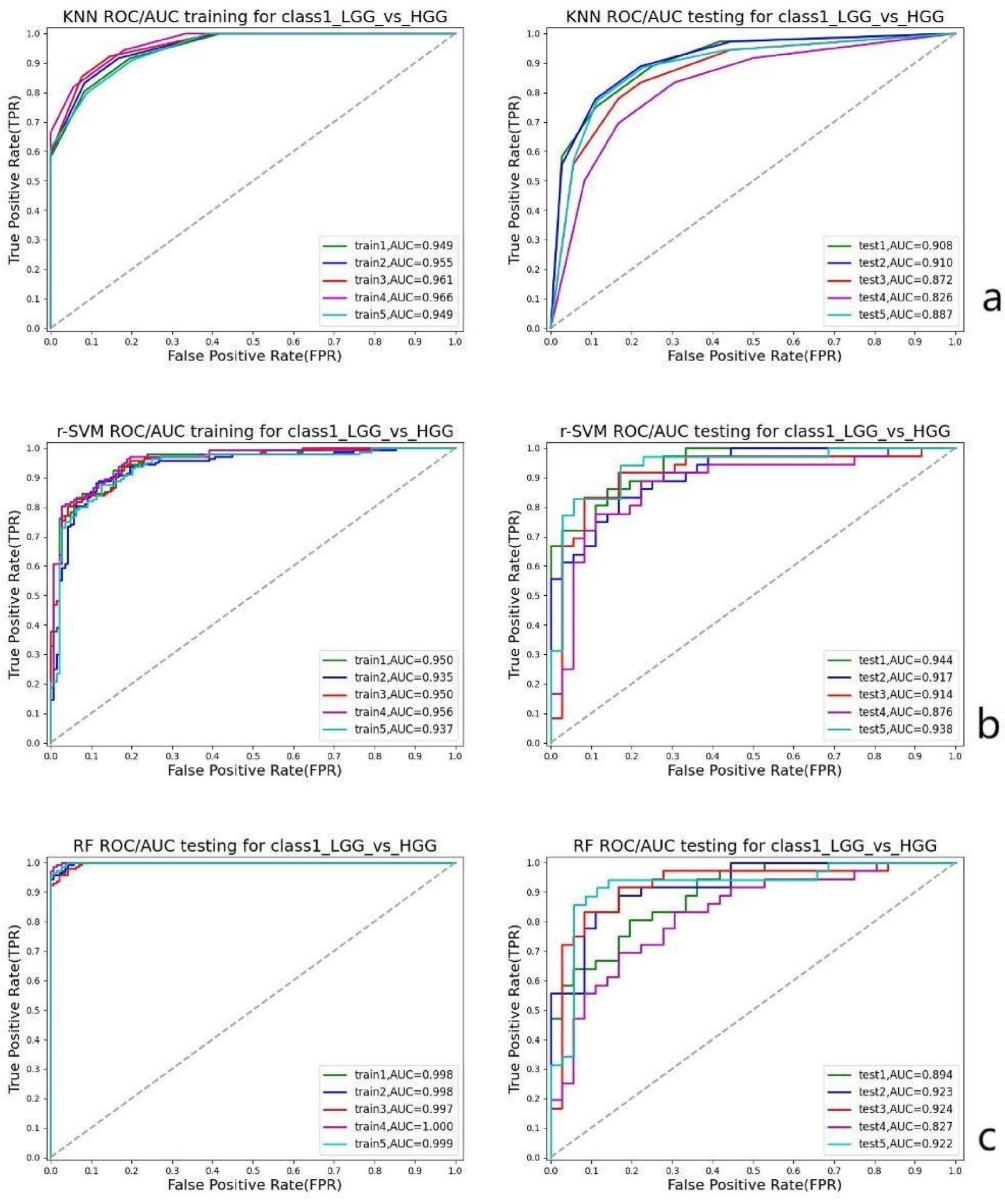
ROC curves for the three classifiers (KNN, r-SVM, and RF) based on five-fold cross-validation. (**a**) KNN; (**b**) r-SVM; (**c**) RF

**Fig. 4:**
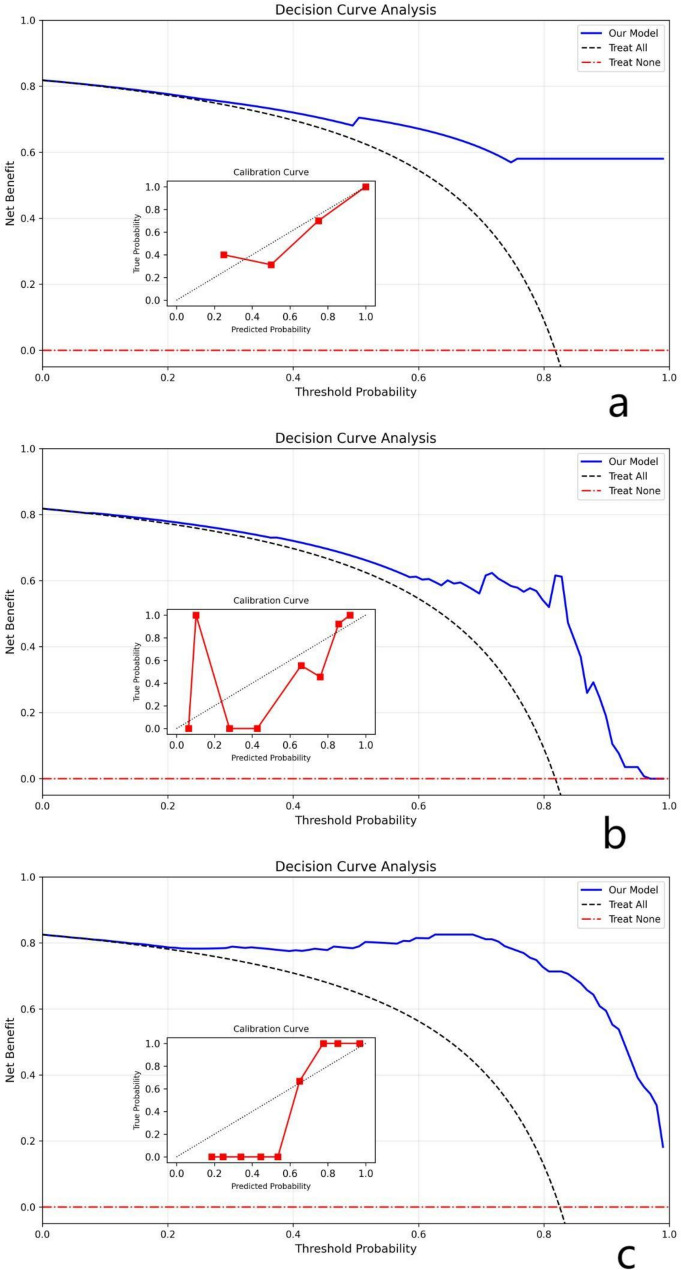
The reliability diagram and decision curves for the three classifiers (KNN, r-SVM, and RF). (**a**) KNN; (**b**) r-SVM; (**c**) RF

Based on the results of 5-fold cross-validation, all three machine learning models demonstrated strong predictive performance in both the training and test cohorts. The r-SVM classifier showed the best performance on the test set, achieving an AUC of 0.92, an accuracy of 0.84(CI: 0.78–0.90), a F1 score of 0.91, a brier score of 0.12 and a log score of 0.39. RF also performed well, with an AUC of 0.90, accuracy of 0.82(CI: 0.75–0.88), a F1 score of 0.89, a brier score of 0.13 and a log score of 0.41. In the decision analysis chart, all three models demonstrated excellent net treatment benefits. However, discrepancies were observed in their calibration curves. The SVM model exhibited significant fluctuations in calibration accuracy across different probability intervals. The RF model showed good calibration performance in medium-to-high prediction probability ranges but performed poorly in low probability zones. The KNN model demonstrated strong consistency between predicted probabilities and actual outcomes on its decision curve, indicating reliable predictive results. The performance metrics of all three classifiers across the cross-validation folds are summarized in Table 3. Figure 3 presents the ROC curves of the classifiers in the test cohort. Figure 4 presents the reliability diagram and decision curve in the test cohort.

**Table 3:** Five-fold cross-validation results for classification performance: low-grade glioma vs. high-grade glioma using KNN, SVM, and RF models

KNN	Accuracy_training	Accuracy_test	lower_test	upper_test	AUC_training	AUC_test	TP_training	TN_training	FP_training	FN_training	TP_test	TN_test	FP_test	FN_test	Sensitivity_training	Sensitivity_test	Specificity_training	Specificity_test	PPV_training	PPV_test	NPV_training	NPV_test	F1_score_training	F1_score_test	Brier_Score_test	log_Score_test	ECE_test
1	0.87	0.83	0.77	0.89	0.95	0.91	109	16	10	8	27	3	3	3	0.93	0.90	0.62	0.50	0.92	0.90	0.67	0.50	0.92	0.90	0.12	1.22	0.03
2	0.89	0.83	0.77	0.89	0.96	0.91	111	16	10	6	28	2	4	2	0.95	0.93	0.62	0.33	0.92	0.88	0.73	0.50	0.93	0.90	0.12	1.22	0.01
3	0.90	0.83	0.77	0.89	0.96	0.87	113	15	10	5	28	2	5	1	0.96	0.97	0.60	0.29	0.92	0.85	0.75	0.67	0.94	0.90	0.15	2.18	0.06
4	0.89	0.72	0.65	0.80	0.97	0.83	110	17	8	8	23	3	4	6	0.93	0.79	0.68	0.43	0.93	0.85	0.68	0.33	0.93	0.82	0.18	3.15	0.08
5	0.88	0.77	0.70	0.84	0.95	0.89	109	17	9	9	25	2	4	4	0.92	0.86	0.65	0.33	0.92	0.86	0.65	0.33	0.92	0.86	0.13	2.19	0.08
min	0.87	0.72	0.65	0.80	0.95	0.83									0.92	0.79	0.60	0.29	0.92	0.85	0.65	0.33	0.92	0.82	0.12	1.22	0.01
mean	0.88	0.80	0.73	0.86	0.96	0.88									0.94	0.89	0.63	0.38	0.92	0.87	0.70	0.47	0.93	0.88	0.14	1.99	0.05

SVM	Accuracy_training	Accuracy_test	lower_test	upper_test	AUC_training	AUC_test	TP_training	TN_training	FP_training	FN_training	TP_test	TN_test	FP_test	FN_test	Sensitivity_training	Sensitivity_test	Specificity_training	Specificity_test	PPV_training	PPV_test	NPV_training	NPV_test	F1_score_training	F1_score_test	Brier_Score_test	log_Score_test	ECE_test
1	0.85	0.86	0.80	0.92	0.95	0.95	117	5	21	0	30	1	5	0	1.00	1.00	0.19	0.17	0.85	0.86	1.00	1.00	0.92	0.92	0.09	0.31	0.08
2	0.89	0.83	0.77	0.89	0.94	0.92	116	11	15	1	30	0	6	0	0.99	1.00	0.42	0.00	0.89	0.83	0.92		0.94	0.91	0.14	0.43	0.12
3	0.85	0.83	0.77	0.89	0.95	0.91	118	4	21	0	29	1	6	0	1.00	1.00	0.16	0.14	0.85	0.83	1.00	1.00	0.92	0.91	0.13	0.41	0.09
4	0.89	0.81	0.74	0.87	0.95	0.87	117	10	15	1	28	1	6	1	0.99	0.97	0.40	0.14	0.89	0.82	0.91	0.50	0.94	0.89	0.14	0.45	0.05
5	0.85	0.86	0.80	0.91	0.94	0.93	117	6	20	1	28	2	4	1	0.99	0.97	0.23	0.33	0.85	0.88	0.86	0.67	0.92	0.92	0.11	0.37	0.15
min	0.85	0.81	0.74	0.87	0.94	0.87									0.99	0.97	0.16	0.00	0.85	0.82	0.86	0.50	0.92	0.89	0.09	0.31	0.05
mean	0.87	0.84	0.78	0.90	0.95	0.92									0.99	0.99	0.28	0.16	0.86	0.84	0.94	0.79	0.93	0.91	0.12	0.39	0.10

RF	Accuracy_training	Accuracy_test	lower_test	upper_test	AUC_training	AUC_test	TP_training	TN_training	FP_training	FN_training	TP_test	TN_test	FP_test	FN_test	Sensitivity_training	Sensitivity_test	Specificity_training	Specificity_test	PPV_training	PPV_test	NPV_training	NPV_test	F1_score_training	F1_score_test	Brier_Score_test	log_Score_test	ECE_test
1	0.97	0.81	0.74	0.87	1.00	0.89	117	21	5	0	28	1	5	2	1.00	0.93	0.81	0.17	0.96	0.85	1.00	0.33	0.98	0.89	0.13	0.38	0.09
2	0.97	0.83	0.77	0.89	1.00	0.92	117	22	4	0	29	1	5	1	1.00	0.97	0.85	0.17	0.97	0.85	1.00	0.50	0.98	0.91	0.12	0.36	0.14
3	0.96	0.83	0.77	0.89	1.00	0.92	118	19	6	0	29	1	6	0	1.00	1.00	0.76	0.14	0.95	0.83	1.00	1.00	0.98	0.91	0.11	0.36	0.11
4	0.99	0.72	0.65	0.80	1.00	0.83	118	23	2	0	25	1	6	4	1.00	0.86	0.92	0.14	0.98	0.81	1.00	0.20	0.99	0.83	0.18	0.58	0.18
5	0.97	0.89	0.83	0.94	1.00	0.92	118	22	4	0	28	3	3	1	1.00	0.97	0.85	0.50	0.97	0.90	1.00	0.75	0.98	0.93	0.10	0.35	0.14
min	0.96	0.72	0.65	0.80	1.00	0.83									1.00	0.86	0.76	0.14	0.95	0.81	1.00	0.20	0.98	0.83	0.10	0.35	0.09
mean	0.97	0.82	0.75	0.88	1.00	0.90									1.00	0.95	0.84	0.22	0.97	0.85	1.00	0.56	0.98	0.89	0.13	0.41	0.13

_training: training cohort; _test: test cohort

lower_test: lower confidence interval of test cohort accuracy

upper_test: upper confidence interval of test cohort accuracy

TP: true HGG; TN: true LGG; FP: false HGG; FN: false LGG; ECE: expected calibration error

## Survival analysis of low-grade (WHO I and II) and high-grade (WHO III and IV) groups

**Fig. 5:**
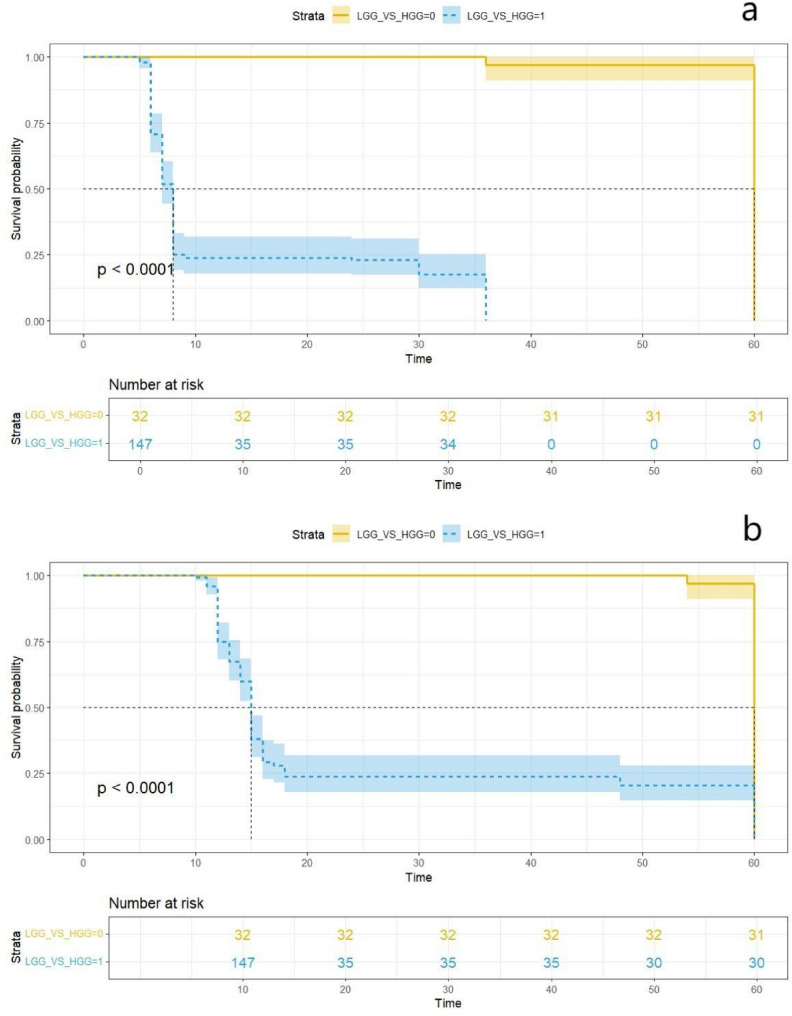
Kaplan-Meier survival curves comparing low-grade and high-grade glioma groups. **a** OS; **b** PFS

The median OS and PFS for the entire cohort were 15 months and 8 months, respectively. Figure 3 illustrates the Kaplan–Meier survival curves for OS and PFS in the low-grade and high-grade groups. Patients in the low-grade group demonstrated significantly longer median OS than those in the high-grade group (60 months vs. 15 months; *p* < 0.0001; Fig. 5a) as well as significantly longer median PFS (60 months vs. 8 months; *p* < 0.0001; Fig. 5b).

## Discussion

The tumor microenvironment in gliomas is often characterized by a sustained state of chronic inflammation, which enables the use of peripheral inflammatory markers to predict tumor behavior and progression [12–14]. MRI remains a critical tool in the diagnosis and evaluation of brain gliomas, with specific parameters such as the rADC and ER serving as important indicators of tumor type and grade [5, 15–17]. In this study, we found that combining radiological features with hematological and inflammatory parameters significantly improved the accuracy of glioma grade prediction. Notably, variables such as age, rADC, monocyte count, LCR, and HALP showed clear discriminatory power between low-grade and high-grade gliomas.

The discriminatory power of these features can be explained by their underlying biological and clinical relevance. Age is a well-established prognostic factor in gliomas, with older patients generally experiencing poorer outcomes [18]. This may be due to age-related changes in the tumor microenvironment, such as increased inflammation and decreased immune surveillance. The rADC, reflecting tumor cellularity and water diffusion, is inversely correlated with glioma grade, with lower rADC values indicating higher cellularity and malignancy [19]. Monocytes, as key components of the innate immune system, contribute to glioma-associated inflammation and angiogenesis, promoting tumor growth and invasion [20]. The lymphocyte-to-C-reactive protein ratio (LCR) and the hemoglobin-albumin ratio-lymphocyte percentage (HALP) are composite inflammatory markers that reflect the balance between systemic inflammation and nutritional status, both of which are known to influence glioma progression [21].

In the tumor microenvironment, glioma pathogenesis involves complex and dynamic interactions between cancer cells and the surrounding stroma, including glial and immune cells. Among the immune components, neutrophils and lymphocytes play a central role in modulating the inflammatory response [1, 22]. In our study, LCR and HALP exhibited significant differences between the high-grade and low-grade glioma groups. These findings are consistent with previous studies demonstrating the prognostic value of systemic inflammatory markers in gliomas [23]. However, our study extends these findings by demonstrating the combined predictive power of LCR and HALP with radiological features, suggesting that a multi-modal approach can provide a more comprehensive assessment of glioma biology.

SVM is a classical supervised learning algorithm. Its core idea is to find an optimal hyperplane (a straight line in two-dimensional space) to maximize the interval between different categories of data points, so as to improve the generalization ability of the model [24]. In this study, the SVM model exhibited superior predictive performance and stability compared with the KNN and RF models in distinguishing between high-grade and low-grade gliomas. This suggests that SVM is well-suited for analyzing complex, high-dimensional datasets and identifying non-linear relationships between features and outcomes.

This study advances current knowledge by demonstrating the feasibility of using a combination of readily available clinical, radiological, and hematological parameters to predict glioma grade with high accuracy. This approach addresses the gap in current clinical practice, where glioma grading relies primarily on invasive surgical biopsy and histopathological evaluation, which are subject to sampling bias and inter-observer variability. Our findings suggest that non-invasive biomarkers can potentially complement or even replace invasive procedures in certain clinical scenarios.

The potential clinical implications of our findings are significant. Accurate preoperative prediction of glioma grade can inform treatment planning, allowing clinicians to tailor therapy to the individual patient’s risk profile. For example, patients with predicted high-grade gliomas may benefit from more aggressive treatment strategies, such as maximal surgical resection followed by adjuvant chemoradiation. Conversely, patients with predicted low-grade gliomas may be managed with less aggressive approaches, such as observation or limited resection, to minimize treatment-related morbidity. Furthermore, our findings may facilitate the development of personalized risk stratification models that can be used to predict prognosis and guide clinical decision-making.

This study has some limitations. First, all patients were recruited from a single institution, which may limit the generalizability of the findings. Second, the retrospective nature of the analysis may introduce selection bias. Additionally, the relatively small number of imaging datasets prevented external validation, which is essential for confirming the robustness and applicability of the proposed models. Future research directions include external validation of our findings in multi-center cohorts, as well as the incorporation of molecular and genetic markers, such as IDH1 mutation status and MGMT promoter methylation, to further refine the predictive accuracy of our models. We also plan to explore the integration of other imaging modalities, such as perfusion MRI and MR spectroscopy, to provide a more comprehensive assessment of glioma biology. Finally, we aim to develop a user-friendly clinical decision support tool that can be used by clinicians to predict glioma grade and guide treatment planning.

## Conclusions

The combination of MRI features and inflammatory markers, analyzed through machine learning models like SVM, holds significant potential for refining glioma prognosis and guiding personalized treatment strategies.

## Electronic supplementary material

Below is the link to the electronic supplementary material.

Supplementary Material 1

## Data Availability

The datasets used and/or analysed during the current study are available from the corresponding author on reasonable request.

## Abbreviations

MRI: Magnetic resonance imaging
ER: Enhancement ratio
ADC: Apparent diffusion coefficient
rADC: Relative ADC
NLR: Neutrophil-to-lymphocyte ratio
CNN: Convolutional neural network
WHO: World Health Organization
CRP: C-reactive protein
dNLR: Derived NLR
PLR: Platelet-to-lymphocyte ratio
PMR: Platelet-to-monocyte ratio
LMR: Lymphocyte-to-monocyte ratio
LCR: Lymphocyte-to-CRP ratio
NCR: Neutrophil-to-CRP ratio
PNI: Prognostic nutritional index
ALI: Advanced lung cancer inflammation index
ALPC: Albumin-lymphocyte-platelet-CRP index
SII: Systemic immune-inflammation index
CAL: CRP-albumin-lymphocyte index
CAR: CRP/albumin ratio
HALP: Hemoglobin-albumin-lymphocyte-platelet index
T2WI: T2-weighted imaging
T1WI: T1-weighted imaging
TR: Repetition time
TE: Echo time
FOV: Field of view
ROI: The region of interest
EI: Enhancement index
ANOVA: The analysis of variance
CI: The confidence interval
kNN: k-nearest neighbors
r-SVM: A radial basis function kernel
RF: Random forest
ROC: Receiver operating characteristic
AUC: ROC curve
OS: Overall survival
PFS: Progression-free survival

## References

[1] Xu G, Li C, Wang Y, Ma J, Zhang J. Correlation between preoperative inflammatory markers, Ki-67 and the pathological grade of glioma. Med (Baltim). 2021, Sept;10;100(36):e26750. 10.1097/MD.0000000000026750. PMID: 34516487; PMCID: PMC8428732. 10.1097/MD.0000000000026750 PMC8428732 34516487

[2] Weng Y, Zhang X, Han J, Ouyang L, Liang M, Shi Z, Liu A, Cai W. Do selected blood inflammatory markers combined with radiological features predict proliferation index in glioma patients? World Neurosurg. 2018, Oct;118:e137–46. 10.1016/j.wneu.2018.06.142. Epub 2018 Jun 26. PMID: 29959082. 29959082 10.1016/j.wneu.2018.06.142

[3] Abdel Razek AAK, Alksas A, Shehata M, AbdelKhalek A, Abdel Baky K, A E-B, Helmy E. Clinical applications of artificial intelligence and radiomics in neuro-oncology imaging. Insights Imag. 2021, Oct, 21;12(1):152. 10.1186/s13244-021-01102-6. PMID: 34676470; PMCID: PMC8531173. 10.1186/s13244-021-01102-6 PMC8531173 34676470

[4] Kumar A, Jha AK, Agarwal JP, Yadav M, Badhe S, Sahay A, Epari S, Sahu A, Bhattacharya K, Chatterjee A, Ganeshan B, Rangarajan V, Moyiadi A, Gupta T, Goda JS. Machine-learning-based radiomics for classifying glioma grade from magnetic resonance images of the brain. J Pers Med. 2023, May, 30;13(6):920. 10.3390/jpm13060920. PMID: 37373909; PMCID: PMC10305272. 37373909 10.3390/jpm13060920 PMC10305272

[5] Bambury RM, Teo MY, Power DG, Yusuf A, Murray S, Battley JE, et al. The association of pretreatment neutrophil to lymphocyte ratio with overall survival in patients with glioblastoma multiforme. J Neurooncol. 2013;114:149–54. 23780645 10.1007/s11060-013-1164-9

[6] Chen F, Chao M, Huang T, Guo S, Zhai Y, Wang Y, Wang N, Xie X, Wang L, Ji P. The role of preoperative inflammatory markers in patients with central nervous system tumors, focus on glioma. Front Oncol. 2022, Nov;22(12):1055783. 10.3389/fonc.2022.1055783. PMID: 36483052; PMCID: PMC9723353. 10.3389/fonc.2022.1055783 PMC9723353 36483052

[7] Dal Bello S, Martinuzzi D, Tereshko Y, Veritti D, Sarao V, Gigli GL, Lanzetta P, Valente M. The present and future of optic pathway glioma therapy. Cells. 2023, Sep, 29;12(19):2380. 10.3390/cells12192380. PMID: 37830595; PMCID: PMC10572241. 37830595 10.3390/cells12192380 PMC10572241

[8] Lin H, Liu C, Hu A, Zhang D, Yang H, Mao Y. Understanding the immunosuppressive microenvironment of glioma: mechanistic insights and clinical perspectives. J Hematol Oncol. 2024, May, 8;17(1):31. 10.1186/s13045-024-01544-7. PMID: 38720342; PMCID: PMC11077829. 38720342 10.1186/s13045-024-01544-7 PMC11077829

[9] Evangelou K, Zemperligkos P, Politis A, Lani E, Gutierrez-Valencia E, Kotsantis I, Velonakis G, Boviatsis E, Stavrinou LC, Diagnostic KA. Therapeutic, and prognostic applications of artificial intelligence (AI) in the clinical management of brain metastases (BMs). Brain Sci. 2025, Jul, 8;15(7):730. 10.3390/brainsci15070730. PMID: 40722321; PMCID: PMC12293690. 40722321 10.3390/brainsci15070730 PMC12293690

[10] Lausch A, Yeung TP, Chen J, Law E, Wang Y, Urbini B, Donelli F, Manco L, Fainardi E, Lee TY, Wong E. A generalized parametric response mapping method for analysis of multi-parametric imaging: a feasibility study with application to glioblastoma. Med Phys. 2017, Nov;44(11):6074–84. 10.1002/mp.12562. Epub 2017 Oct 13. PMID: 28875538. 28875538 10.1002/mp.12562

[11] Salari E, Chen X, Wynne JF, Qiu RLJ, Roper J, Shu HK, Yang X. Prediction of early recurrence of adult-type diffuse gliomas following radiotherapy using multi-modal magnetic resonance images. Med Phys. 2024, Nov;51(11):8638–48. 10.1002/mp.17382. Epub 2024 Sep 2. PMID: 39221589; PMCID: PMC11530302. 39221589 10.1002/mp.17382 PMC11530302

[12] Lin ZX. Glioma-related edema: new insight into molecular mechanisms and their clinical implications. Chin J Cancer. 2013;32:49–52. 23237218 10.5732/cjc.012.10242 PMC3845586

[13] Wang XF, Lin GS, Lin ZX, Chen YP, Chen Y, Zhang JD, et al. Association of pSTAT3-VEGF signaling pathway with peritumoral edema in newly diagnosed glioblastoma: an immunohistochemical study. Int J Clin Exp Pathol. 2014;7:6133–40. 25337261 PMC4203232

[14] Jensen RL, Ragel BT, Whang K, Gillespie D. Inhibition of hypoxia inducible factor-1alpha (HIF-1alpha) decreases vascular endothelial growthfactor (VEGF) secretion and tumor growth in malignant gliomas. J Neurooncol. 2006;78:233–47. 16612574 10.1007/s11060-005-9103-z

[15] Barajas RF Jr, Hodgson JG, Chang JS, Vandenberg SR, Yeh RF, Parsa AT, et al. Glioblastoma multiforme regional genetic and cellularexpression patterns: influence on anatomic and physiologic MR imaging. Radiology. 2010;254:564–76. 20093527 10.1148/radiol.09090663 PMC2809924

[16] Niu L, Feng WH, Duan CF, Liu YC, Liu JH, Liu XJ. The value of enhanced MR radiomics in estimating the IDH1 genotype in high-grade gliomas. Biomed Res Int. 2020, Oct;24(2020):4630218. 10.1155/2020/4630218. PMID: 33163535; PMCID: PMC7604586. 10.1155/2020/4630218 PMC7604586 33163535

[17] Cui Y, Dang Y, Zhang H, Peng H, Zhang J, Li J, Shen P, Mao C, Ma L, Zhang L. Predicting isocitrate dehydrogenase genotype, histological phenotype, and Ki-67 expression level in diffuse gliomas with an advanced contrast analysis of magnetic resonance imaging sequences. Quant Imag Med Surg. 2023, Jun, 1;13(6):3400–15. 10.21037/qims-22-887. Epub 2023 May 15. PMID: 37284074; PMCID: PMC10240019. 10.21037/qims-22-887 PMC10240019 37284074

[18] Yeo KK, Burgers DE, Brodigan K, Fasciano K, Frazier AL, Warren KE, Reardon DA. Adolescent and young adult neuro-oncology: a comprehensive review. Neurooncol Pract. 2021, Feb, 18;8(3):236–46. 10.1093/nop/npab001. PMID: 34055371; PMCID: PMC8153805. 34055371 10.1093/nop/npab001 PMC8153805

[19] Foltyn-Dumitru M, Schell M, Sahm F, Kessler T, Wick W, Bendszus M, Rastogi A, Brugnara G, Vollmuth P. Advancing noninvasive glioma classification with diffusion radiomics: exploring the impact of signal intensity normalization. Neurooncol Adv. 2024, Mar, 22;6(1): vdae043. 10.1093/noajnl/vdae043. PMID: 38596719; PMCID: PMC11003539. 10.1093/noajnl/vdae043 PMC11003539 38596719

[20] Broekman ML, Maas SLN, Abels ER, Mempel TR, Krichevsky AM, Breakefield XO. Multidimensional communication in the microenvirons of glioblastoma. Nat Rev Neurol. 2018, Aug;14(8):482–95. 10.1038/s41582-018-0025-8. PMID: 29985475; PMCID: PMC6425928. 29985475 10.1038/s41582-018-0025-8 PMC6425928

[21] Liu XY, Zhang X, Zhang Q, Ruan GT, Xie HL, Liu T, Song MM, Ge YZ, Deng L, Shi HP. Lymphocyte-C-reactive protein ratio with calf circumference could better predict survival of patients with non-metastatic cancer. Sci Rep. 2023, May, 3;13(1):7217. 10.1038/s41598-023-34096-w. PMID: 37137949; PMCID: PMC10156854. 37137949 10.1038/s41598-023-34096-w PMC10156854

[22] Khalili N, Kazerooni AF, Familiar A, Haldar D, Kraya A, Foster J, Koptyra M, Storm PB, Resnick AC, Nabavizadeh A. Radiomics for characterization of the glioma immune microenvironment. NPJ Precis Oncol. 2023, Jun, 19;7(1):59. 10.1038/s41698-023-00413-9. PMID: 37337080; PMCID: PMC10279670. 37337080 10.1038/s41698-023-00413-9 PMC10279670

[23] Yang C, Wen HB, Zhao YH, Huang WH, Wang ZF, Li ZQ. Systemic inflammatory indicators as prognosticators in glioblastoma patients: a comprehensive meta-analysis. Front Neurol. 2020, Oct;7(11):580101. 10.3389/fneur.2020.580101. PMID: 33117267; PMCID: PMC7575748. 10.3389/fneur.2020.580101 PMC7575748 33117267

[24] Huang MW, Chen CW, Lin WC, Ke SW, Tsai CF. SVM and SVM ensembles in breast cancer prediction. PLoS One. 2017, Jan, 6;12(1):e0161501. 10.1371/journal.pone.0161501. PMID: 28060807; PMCID: PMC5217832. 28060807 10.1371/journal.pone.0161501 PMC5217832

