# Supplementary material for: Predictive power of combined inflammatory markers and magnetic resonance imaging features for glioma grading using machine learning: a retrospective study
Source: BMC Med Imaging. 2025 Oct 21;25:421. doi: 10.1186/s12880-025-01946-0 (PMC12542063; doi:10.1186/s12880-025-01946-0)
Supplement: Supplementary file 1 — Supplementary Material 1 [file 12880_2025_1946_MOESM1_ESM.docx]

| **Inflammatory markers** | **Formula** | **Clinical significance** |
| --- | --- | --- |
| leucocyte count(WBC) | Total white blood cell (number) | Reflects the overall inflammatory and immune response. |
| Neutrophile granulocyte (NE) | Total neutrophils (number) | Used to assess the presence and severity of infection or inflammation. |
| leukomonocyte (L) | Total lymphocyte count (number) | Reflecting the state of immunity, low levels may indicate immunosuppression. |
| platelet (Plt) | Platelet total count (number) | Reflects coagulation and inflammatory status, elevated during inflammation. |
| Ratio of lymphocytes to monocytes (LMR) | Lymphocyte to monocyte ratio (LMR = L/M) | High values were associated with better immune status and prognosis. |
| Ratio of lymphocytes to C-reactive protein (LCR) | Lymphocyte to C-reactive protein ratio (LCR = L / CRP) | May reflect inflammatory response and immunosuppression. |
| Ratio of neutrophils to lymphocytes (NCR) | Neutrophils to lymphocytes ratio (NCR = N/L) | High values indicate systemic inflammation and poor prognosis. |
| prognosis nutrition index (PNI) | Prognostic Nutritional Index (PNI = albumin + 5 x L) | Assessment of nutritional and immune status was associated with survival outcomes. |
| Improved neuroinflammatory marker ratio (dNLR) | Neutrophils/ [white blood cell count-neutrophils] (dNLR = N / [WBC-N]) | Reflects the degree of inflammation and evaluates the ability to respond to immune responses. |
| Albumin to lymphocyte ratio (ALPC) | Albumin to lymphocyte ratio (ALPC = Alb/L) | Reflects overall systemic inflammation and immunosuppression. |
| Systemic immune inflammation index (SII) | Systemic immune inflammation index (SII = Plt × N/L) | Reflects systemic inflammatory response and immune function. |
| Calcium to lactic acid ratio (CAL) | Calcium to lactic acid ratio（CAL = Ca / Lactate） | Associated with metabolic responses to inflammation. |
| Ratio of C-reactive protein to albumin (CAR) | Ratio of C-reactive protein to albumin (CAR = CRP / Alb) | High values indicate severe inflammation and poor prognosis. |
| Hemoglobin-albumin-lymphocyte-platelet index (HALP) | Hemoglobin albumin lymphocyte platelet index (HALP = Hb x Alb x L/Plt) | Systemic inflammation and nutritional status markers in cancer patients. |

Appendix Inflammatory markers
